# Transmission of Hepatitis A Virus through Combined Liver–Small Intestine–Pancreas Transplantation

**DOI:** 10.3201/eid2304.161532

**Published:** 2017-04

**Authors:** Monique A. Foster, Lauren M. Weil, Sherry Jin, Thomas Johnson, Tonya R. Hayden-Mixson, Yury Khudyakov, Pallavi D. Annambhotla, Sridhar V. Basavaraju, Saleem Kamili, Jana M. Ritter, Noele Nelson, George Mazariegos, Michael Green, Ryan W. Himes, David T. Kuhar, Matthew J. Kuehnert, Jeffrey A. Miller, Rachel Wiseman, Anne C. Moorman

**Affiliations:** Centers for Disease Control and Prevention, Atlanta, Georgia, USA (M.A. Foster, T.R. Hayden-Mixson, Y. Khudyakov, P.D. Annambhotla, S.V. Basavaraju, S. Kamili, J.M. Ritter, N. Nelson, D.T. Kuhar, M.J. Kuehnert, J.A. Miller, A.C. Moorman);; Texas Department of State Health Services, Austin, Texas, USA (L.M. Weil);; Harris County Public Health and Environmental Services, Houston, Texas, USA (S. Jin);; Houston Health Department, Houston (T. Johnson);; Children’s Hospital of Pittsburgh of University of Pittsburgh Medical Center, Pittsburgh, Pennsylvania, USA (G. Mazariegos, M. Green);; Texas Children’s Hospital, Houston, (R.W. Himes);; Pennsylvania Department of Health, Harrisburg, Pennsylvania, USA (J.A. Miller);; Texas Department of State Health Services, Austin (R. Wiseman)

**Keywords:** hepatitis A virus, transplantation, viremia, viruses, healthcare workers, transmission, liver, small intestine, pancrease

## Abstract

Although transmission of hepatitis A virus (HAV) through blood transfusion has been documented, transmission through organ transplantation has not been reported. In August 2015, state health officials in Texas, USA, were notified of 2 home health nurses with HAV infection whose only common exposure was a child who had undergone multi–visceral organ transplantation 9 months earlier. Specimens from the nurses, organ donor, and all organ recipients were tested and medical records reviewed to determine a possible infection source. Identical HAV RNA sequences were detected from the serum of both nurses and the organ donor, as well as from the multi–visceral organ recipient’s serum and feces; this recipient’s posttransplant liver and intestine biopsy specimens also had detectable virus. The other organ recipients tested negative for HAV RNA. Vaccination of the donor might have prevented infection in the recipient and subsequent transmission to the healthcare workers.

## Abstract 2

Vaccination of the donor might have prevented infection in the recipient and subsequent transmission to healthcare workers.

Hepatitis A virus (HAV), the most common cause of viral hepatitis, is a nonenveloped RNA virus belonging to the family *Picornaviridae* (*1*,*2*). Approximately 1.5 million clinical cases of HAV occur worldwide annually; the virus is commonly spread person to person through the fecal–oral route (*2*). Although rates of HAV infection have declined by 95% in the United States since a vaccine became available, infections continue to result from close personal contact with an infected household member or common-source outbreaks from contaminated food or water (*3*). HAV can cause relapsing and fulminant hepatitis, but fatal infection is rare.

Parenteral transmission of HAV through contaminated blood products or needles is also rare, despite the presence of viremia up to 30 days before symptom onset (*4*). No screening tests for HAV infection are required for blood, organ, or tissue donation in the United States (*5*). HAV transmission through solid-organ transplantation has not been reported in the literature.

In August 2015, genetically identical HAV was recovered from 2 healthcare workers (HCWs) participating in the care of a child recipient of multiple visceral organs. To prevent infection in other organ recipients and contacts, the Centers for Disease Control and Prevention (CDC), along with state and local health departments, conducted an investigation to determine the source of the HCW infection and whether HAV was transmitted through the solid-organ transplantation.

## Case Report

In August 2015, the Texas Department of State Health Services received requests from 2 separate local health departments for postexposure prophylaxis recommendations for contacts of 2 nurses with confirmed HAV infection. In both nurses, symptomatic infections developed within days of each other; both nurses worked for the same pediatric home healthcare agency. The nurses did not have shared exposures other than 1 patient and had not been vaccinated for hepatitis A.

The shared patient was a 7-year-old who underwent multiorgan (liver, small bowel, and pancreas) transplantation because of megacystis microcolon intestinal hypoperistalsis syndrome, a rare congenital condition characterized by a largely dilated nonobstructed urinary bladder (megacystis), very small colon (microcolon), and decreased or absent intestinal movements (intestinal peristalsis). The transplantation occurred in December 2014.

This patient’s posttransplant course was complicated by intraabdominal abscesses, acute liver rejection, Epstein-Barr virus enteritis, cytomegalovirus infection, and lymphoproliferative disorder. The patient was discharged to home in March 2015 with an alanine aminotransferase (ALT) level of 49 IU/L (referemce 0–50 IU/L). By the following month, ALT had increased to 324 IU/L, and by June 2015, to 515 IU/L. During the time of ALT increase, the patient, who had a colostomy and ileostomy, also had increased stoma output. Because of worsening clinical symptoms, a liver biopsy was conducted on June 19, 2015; results showed features of moderate acute cellular rejection, as well as diffuse, lobular acute and chronic inflammation. On June 30, a repeat liver biopsy was performed because of persistent ALT elevation despite increased treatment for rejection.

The multi–visceral organ recipient had received 2 doses of hepatitis A vaccine, as part of routine childhood vaccinations, and was IgG HAV positive in 2013, indicating prior immunity against HAV, although the patient was immune suppressed after transplantation. Because of the other clinical conditions that could have explained the ALT elevation and gastrointestinal symptoms, HAV infection was initially not considered. When subsequent testing prompted by the infections in the recipient’s caregivers revealed the multi–visceral organ recipient was positive for HAV RNA, a laboratory and epidemiologic investigation focused on whether infection was due to recent fecal–oral transmission or from solid-organ transplantation. Available transplant-related specimens were tested to rule out transmission through transplantation.

## Methods

### Clinical and Epidemiologic Review

The case was reported to the Organ Procurement and Transplantation Network and reviewed by the Network’s ad hoc Disease Transmission Advisory Committee as a suspected donor-derived disease event. Through representation on advisory committee, CDC, with support from state and local health departments, investigates potential transmission to other organ recipients. To determine whether the multi–visceral organ recipient acquired HAV infection through transplantation and to identify other potentially infected HCWs, public health investigators reviewed medical records, interviewed household contacts, and conducted additional case finding among healthcare providers and other transplant recipients. This investigation included review of records from the home health agency that employed both home health nurses; consultation with occupational health staff from the treating facilities; and review of surveillance data from the jurisdictions in which the patient received care.

### Laboratory Specimen Collection and Testing

Laboratory testing was conducted at the laboratory of CDC’s Division of Viral Hepatitis, National Center for HIV, Viral Hepatitis, STD, and TB Prevention (Atlanta, GA, USA). IgG HAV, IgM HAV, and HAV RNA extraction of serum was conducted on both HCWs; the organ donor; and recipients of the visceral organs, heart, and kidneys. When HAV RNA was detected from serum samples, PCR and phylogenetic analysis was conducted to determine relatedness. Similar testing was conducted on frozen and paraffin-embedded liver and small bowel tissue biopsy specimens from the multi–visceral organ recipient. Methods of viral extraction from paraffin-embedded tissue have been described previously (*6*). All HAV RNA–positive samples were used to sequence the HAV VP1/P2B (viral protein 1/amino terminus of 2B ) genomic region, and phylogenetic analysis was performed by comparing these sequences with archived HAV sequences contained within the CDC HAV sequence database (*7*).

## Results

### Results of Epidemiologic Investigation

Case investigations revealed that, during their incubation and infectious periods, the home health nurses cared for a total of 12 children. However, they had only 1 patient in common: the multi–visceral organ recipient (*8*).

**Figure 1:**
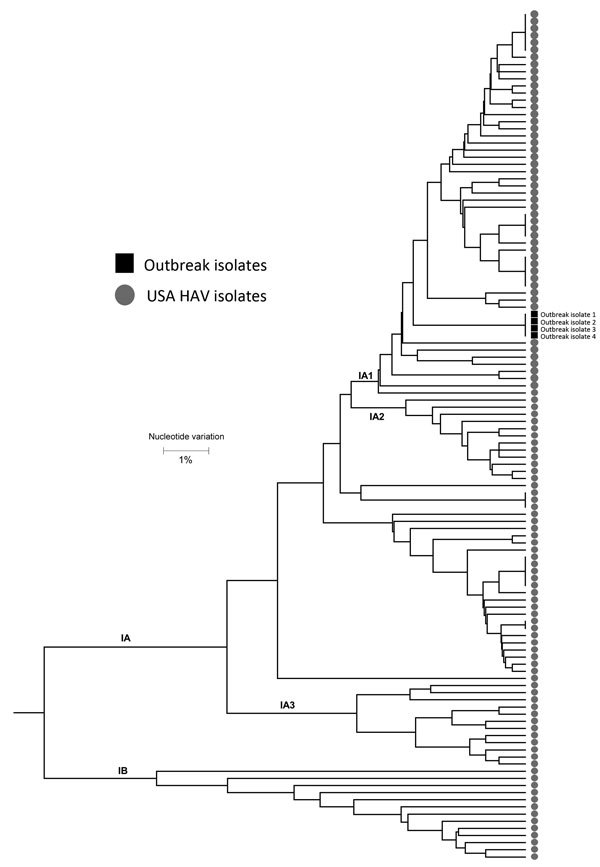
Polygenetic analysis of HAV isolates within the Centers for Disease Control and Prevention’s US HAV database. The genomic regions are the 315-bp long HAV VP1/P2B (viral protein 1/amino terminus of 2B). Black squares indicate isolates from the outbreak of HAV transmitted through a combined liver–small intestine–pancreas transplantation, Texas, USA, 2014–2015. Scale bar indicates nucleotide variation. HAV, hepatitis A virus.

An additional HCW with HAV infection was identified during a case-contact interview with the mother of the multi–visceral organ recipient. This nurse also provided care to the multi–visceral organ recipient, and jaundice, diarrhea, and arthralgia later developed that required inpatient admission. HAV infection was diagnosed by serologic testing during that hospitalization; this nurse also had not received a hepatitis A vaccination. Care for the multi–visceral organ recipient provided by all 3 nurses included managing watery feces (e.g., changing diapers and ostomy bags). Epidemiologic investigation and the resulting timeline (Figure 1) provide evidence that all 3 nurses most likely were infected by exposure to the multi–visceral organ recipient.

The organ donor, an 8-year-old who died in a motor vehicle collision, traveled to Guatemala, a country to which HAV is endemic, 6 months before death. In addition to the visceral organs, which were transplanted into the index patient, the heart and both kidneys were transplanted into 3 other recipients. The organ donor’s name was found in a vaccination registry maintained by 1 of the states of residence, which indicated that the hepatitis A vaccination was not given. Serum banked from donation was negative for evidence of antibody to hepatitis B surface antigen, further indicating that the donor might not have completed recommended childhood vaccinations against any viral hepatitis.

### Results of Laboratory Investigation

**Figure 2:**
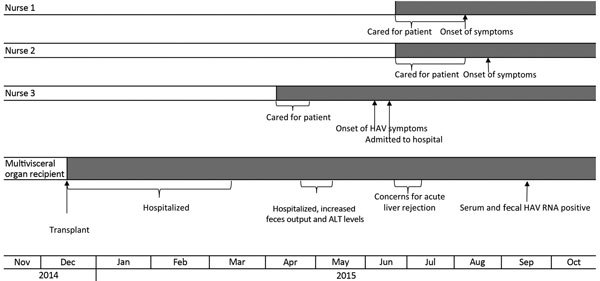
Clinical timeline of HAV infection among a multi–visceral organ transplantation recipient and infected healthcare workers, Texas, 2014–2015. ALT, alanine aminotransferase; HAV, hepatitis A virus.

PCR results for HAV RNA of serum specimens from the 2 home health nurses and the multi–visceral organ recipient are shown in Table 1. The third HCW had recovered by the time the outbreak was identified and had no available specimen from when she was symptomatic. The specimens from the 2 nurses and the organ recipient had detectable HAV RNA with sequences genetically identical to those of other isolates in the CDC HAV isolate database, thus confirming the multi–visceral organ recipient as the source of the HCW infections (Figure 2).

**Table 1:** Laboratory results for case-patients and contacts related to HAV outbreak, Texas, 2015*

Patient or source of specimen	Outcome or status	HAV rRT -PCR	Serologic testing		Vaccination status
			IgM	IgG	
Donor	Deceased	Detected	Detected	Not detected	Unvaccinated
Multi–visceral organ recipient	Persistent infection	Detected	Detected	Detected	Vaccinated
Heart recipient	Immune	Not detected	Not detected	Detected	Vaccinated
Left kidney recipient	Immune	Not detected	Not detected	Detected	Vaccinated
Right kidney recipient	Immune	Not detected	Not detected	Detected	Vaccinated
Home health nurse A	Recovered	Detected	Detected	Not detected	Unvaccinated
Home health nurse B	Recovered	Detected	Detected	Not detected	Unvaccinated
Inpatient nurse	Recovered	No sample	Detected	Not detected	Unvaccinated

*HAV, hepatitis A virus; rRT-PCR, real-time reverse transcription PCR.

Retrospective testing of banked liver biopsy tissue from the multi–visceral organ recipient showed no detectable HAV RNA in native liver but detectable HAV RNA in all subsequent samples, as early as 5 days after transplantation. Sequential serum and fecal specimens from the multi–visceral organ recipient were positive for HAV RNA through January 2016; follow-up serum specimens drawn monthly during March–May 2016 and a fecal specimen from May 2016 showed clearance of HAV in serum but persistent low-level viremia in feces (Table 2). Frozen serum specimens from the 8-year-old organ donor also were positive for HAV RNA (Table 1). Serum specimens collected from the heart and kidney recipients ≈10 months after transplant tested negative for HAV RNA at CDC and negative for HAV IgM at their sites of clinical care (Table 1). These recipients also had no clinical signs or symptoms consistent with HAV infection.

**Table 2:** Pathologic, serum, and fecal HAV RNA quantification test results of multi–visceral organ recipient, Texas, 2014–2015*

Date collected	Liver HAV RNA, IU/mL	Small bowel HAV RNA, IU/mL	Serum HAV RNA, IU/mL	Fecal HAV RNA, IU/mL
2014 Dec 18	Native liver, none detected			
2014 Dec 23	34,000			
2015 Jan 23	1,500,00			
2015 Feb 10		7,000		
2015 Mar 10		5,400		
2015 Apr 16	>90,000,000			
2015 Sep 21			>90,000,000	>90,000,000
2015 Sep 29			746,000,000	>90,000,000
2015 Oct 15			1,400,000	372,000
2015 Nov 9			27,900	
2015 Nov 16			17,300	
2015 Nov 23			13,000	1,500,000
2015 Dec 3			38,900	51,900
2016 Jan 11			68,000	22,600
2016 Mar 9				320
2016 Apr 6			Not detected	270
2016 May 4			Not detected	Not detected

*Blank cells indicate that no specimen was available for testing. HAV, hepatitis A virus.

## Discussion

The isolation of genetically identical HAV RNA sequences from the multi–visceral organ recipient and the organ donor indicates HAV infection was transmitted through organ transplantation, even though the index patient had earlier evidence of immunity. The 3 HCWs associated with this investigation most likely were infected by the traditional fecal–oral route. The infectious period of the multi–visceral organ recipient is also among the longest documented in a person infected with HAV, typically an acute disease (*9*).

Diagnosis in the multi–visceral organ recipient probably was delayed due to deferred testing because of concurrent conditions that provided alternative explanations for the recipient’s clinical presentation and history of prior HAV immunity. The patient had history of vaccination but immune suppression probably blunted antibody response. Symptomatic persons typically show elevated ALT levels that coincide with onset of clinical illness. The infected organ recipient had elevated ALT shortly after liver transplantation, but it coincided with other viral infections (Epstein-Barr virus and cytomegalovirus) and was later assumed to be related to possible acute liver rejection. 

The process that results in prolonged courses of HAV infection is unknown (*10*). In contrast to hepatitis B and C viruses, HAV is not typically associated with a prolonged infectious carrier state (*11*). Nonetheless, this case report, and scant longitudinal studies in the literature, demonstrate the potential for ongoing transmission. In published longitudinal studies, HAV viremia persisted for median periods of 22–42 days in immunocompetent persons and 256–490 days in immunocompromised persons (*11*,*12*). Similar findings have been published of persistent viral shedding in feces with median days of detection after symptom onset of 81–127 days (*11*). The level and length of HAV infection and fecal shedding make the carrier a potential continuing infectious source of the virus, which occurred in this case.

Because the primary transmission of HAV is fecal–oral, it is not surprising that diarrhea or fecal incontinence leads to the spread of infection (*13*). Nosocomial outbreaks are uncommon because hygienic practices are generally adhered to more consistently when the patient is symptomatic enough to be hospitalized (*14*). The use of contact precautions is recommended for HCWs caring for patients with HAV who are diapered or incontinent (*15*). Because HCWs do not have increased prevalence of HAV infection and because nosocomial outbreaks of HAV are rare, hepatitis A vaccination is not mandatory for HCWs in the United States (*16*,*17*). In this case, the multi–visceral organ recipient’s ileostomy and colostomy output had increased, but it was difficult to determine whether these increases represented symptoms of HAV infection because her stoma output was typically described as a continuous liquid, even before hepatitis developed. Also, the home health nurses were spending 12 hours per shift inside the patient’s home, where they ate meals and shared space with the patient’s family, and thus were considered household contacts, which increased their risk for infection. Previous studies have shown that length of contact with an infected patient increases the attack rate of HCWs, and postexposure prophylaxis is recommended for household contacts of infected patients (*14*,*16*). The parents of the organ recipient were tested for HAV infection before the patient was determined to be the source. The mother’s test results indicated immunity to HAV, and the father was not HAV immune but received hepatitis vaccination at testing. Once the infection was detected, contact precautions were instituted during subsequent hospitalizations, the local health department recommended the patient’s home health nurses be fully vaccinated against HAV, and no further transmissions to HCWs were detected during the subsequent 8 months of continued viral shedding in the patient’s feces.

Transmission of HAV after blood transfusion has been established previously but has not been reported through organ transplantation (*5*). Organ procurement organizations typically complete a medical history questionnaire focusing on prior vaccinations, infections, and exposures to screen donors and recipients for multiple types of infection (*18*). Because of the acute nature of HAV infection, pretransplant testing is not routinely done, although hepatitis A vaccination of transplantation candidates is recommended (*16*). The multi–visceral organ recipient was vaccinated, but studies have shown loss of immunity to HAV after transplantation because of immunosuppression (*19*,*20*). The heart and kidney transplant recipients had evidence of immunity and probably were protected through previous vaccination. It is unknown whether the differences in immunosuppression and types of organs transplanted in the heart and kidney recipients were also factors in preventing HAV infection after transplantation in these persons.

In 2006, the Advisory Committee on Immunization Practices recommended routine hepatitis A vaccination for all children beginning at 12–23 months of age (*16*). Both inactivated whole-virus vaccines available in the United States are well tolerated and effective, showing serologic levels of protection for at least 17 years (*21*,*22*). In 2014, however, 2-dose vaccination coverage among children 19–35 months of age in the United States was only 57.5%, the lowest vaccine coverage for a complete vaccine series among the routine childhood vaccines (*23*). The 8-year-old organ donor in this report most likely was not vaccinated against HAV, and transmission to the recipient and HCWs could have been prevented had vaccination occurred.

Rapid communication between public health officials, physicians, transplant centers, and organ procurement organizations made locating and testing the other organ recipients in this investigation possible. Because of the low US incidence of HAV infection and typically brief self-limiting course of disease, an HAV-unvaccinated organ donor is unlikely to be acutely infected at death and transmit HAV to a patient. The acute nature of HAV infection, low population HAV infection rate, and low rate of HAV infection–associated hospitalization make universal vaccination of HCWs and pretransplant testing for patients impractical. Vaccination against hepatitis A of the organ donor at 12–23 months of age, as recommended by the Advisory Committee on Immunization Practices, most likely would have prevented infection of the multi–visceral organ recipient and exposed HCWs.
